# Topics of Public Interest

**Published:** 1912-12

**Authors:** 


### TOPICS OF PUBLIC INTEREST.

---

Deaths from Tuberculosis number about 35,000 annually in the U. S. We quote this statement from an exchange and beg leave to call attention to the fact that the Census Report for 1908, ascribes over 78,000 deaths to this cause, for the registration area which, at that time, embraced about 51 per cent. of the total population. When facts are so easily obtainable, is there any excuse for publishing an underestimate of so gross a degree—between a quarter and a fifth of the true figures?

THE LANE MEDICAL LIBRARY of Leland Stanford, Jr., University, was dedicated Nov. 3. It is located at Sacramento and Webster Sts., San Francisco.

---

CROPS OF THE UNITED STATES.

| | Million Bushels 1912 | Million Bushels 1911 |
|---|---|---|
| Wheat | 720 | 621 |
| Corn | 3,169 | 2,531 |
| Oats | 1,417 | 922 |
| Potatoes | 414 | 292 |

Other staples have shown a corresponding increase, which should make prices lower and life less strenuous.

---

LUNACY EXAMINATION AND AUTOPSY FEES.—The Elmira Academy of Medicine has established a minimum rate of $15. The minimum fee for an autopsy has been set at $20. In our experience payment for autopsy is rare, except in medico-legal cases. In private cases the physician usually has to plead for the privilege of making it free and we recall a case in which the sister of a charity patient demanded to be paid for her consent. Arguing that a woman who would try to make money out her sister's dead body would be guilty of any crime, we thereupon demanded an autopsy to show that death was due to natural causes.

---

STERILIZATION OF CRIMINALS IN OREGON—The Supreme Court has recently, on a test case, upheld the constitutionality of the law.

---

MEDICAL CERTIFICATE PRELIMINARY TO ISSUANCE OF MARRIAGE LICENSE.—The Indiana Board of Health expects that this will be required by law within a year.

---

INTRODUCTION OF AIR PER RECTUM.—A Michigan City, Ind., workman has recently been killed by this form of practical joke. A similar case in the Atlas Engine Works of Indianapolis, several years ago, terminated in recovery. Dr. Willoughby Walling of Louisville narrated a case of rupture of the colon by water introduced from a hand syringe. (A. W. B. in *Ind. Med. Jour.*, Oct., 1912.

---

THE HIGGINS MEMORIAL HOSPITAL of Olean, was dedicated Nov. 2.

THE PUBLIC SERVICE COMMISSION is after the electric companies. For Buffalo, it has adduced evidence that free service has been extended to officials and that the rates vary. There is no place in the world where cheap and abundant water power and communities of sufficient size for economic distribution are united as in western New York. Only the wealthy can afford electric light—not to mention heat—in the frontier cities, yet many small villages, where there can be no economy of wholesale distribution and where power must be produced from coal bought in small quantities and at necessarily high freight rates, furnish electricity so cheap that tenants of fifteen dollar cottages burn it all day and all night on their verandahs. The abuse of privileges by corporations, in so flagrant a manner, fosters socialism. The substitution of electric light and heat for that derived from combustion, is a matter of public safety and public health, as well as of individual convenience.

---

THE METRIC CARAT FOR DIAMONDS, 200 milligrams, is to be adopted in America. The present carat weighs about 205 milligrams. Possibly, if the meter, liter and kilogram were respectively smaller instead of larger than the yard, quart and 2 pound weight, there would not be so much opposition to their introduction.

---

STERILIZATION OF WATER BY ZINC. Diehner, a French scientist, claims that a piece of zinc in water, or the zinc surface of a galvanized pail, kills bacteria, probably by virtue of their secretion of some acid, while not enough zinc is liberated to be toxic. It is well known that bacteria die on any clean metallic surface.

---

PENSIONERS IN 1912, amount to 538 million soldiers and sailors, 322 million dependents, including widows, and 362 army nurses. The appropriation is 151 1-2 million dollars, a decrease of 3 millions from last year.

---

PEACE HATH HER HEROES NO LESS THAN WAR. 14 surgeons of the U. S. Marine Hospital and Public Health Service, at which army and navy men used to sneer, as a civilian service, have contracted yellow fever; 11 have died of tuberculosis, one of cholera Asiatica.

---

VICTIMS OF AN OVERCROWDED PROFESSION would do well to remember that there is an imperative call for medical missionaries, especially in China, but in various other countries.

Electric Warning of Enuresis. Genouville of France has invented an ingenious device. The terminals of a battery—a wet cell would be more appropriate but we presume any form could be used—are connected with an electric bell via the diaper or a pad which, on becoming moistened with the natural saline solution, conducts the current and causes the bell to ring. Yankee ingenuity is rather a misnomer. While, in many respects, epoch making discoveries have been due to Americans, there are many exceptions and, in Europe, one finds the practical development of inventions much more rapid and general, because they are quickly reduced to a reasonable price.

---

Poliomyelitis Infection is considered by the Springfield, Mass., board of health to be carried by cats.

---

Failure of Homoeopathic Treatment. A workingman killed by a current of 3500 volts at West Lynn, Mass., was subjected to a current of 50000 volts in the hope of resuscitating him, but without success. It is not stated, however, that this was done by a homoeopathic practitioner and it must be conceded that an "allopathic" dose was used.

---

Similia Similibus. One of our contemporaries accuses Elbert Hubbard of vulgarity, employing a metaphor that impresses us as about as vulgar as anything the Philistine ever published, excepting the reference to the Genesee Hunting Club and to Taft and the Trusts. Another exchange prints a five-page diatribe on Hubbard's antagonism to vaccination a waste of ammunition.

---

Preservation of Rubber. Michailowsky announces that sprinkling with naphthalin, kept rubber tubing in a glass jar, in good condition for three years and states that the method is available for all kinds of soft rubber goods.

---

The American Surgical Association has appointed a committee consisting of Drs. William L. Estes, South Bethlehem, Pa.; Thomas W. Huntingdon, San Francisco, California; John B. Walker, New York City; Edward Martin, Philadelphia; and John B. Roberts, Chairman, 313 S. 17th Street, Philadelphia, to report on the operative and non-operative treatment of closed and open fratcures of the long bones and the value of radiography in the study of these injuries. Surgeons, who have published papers relating to this subject within the last ten years, will confer a favor by sending two reprints to the chairman of the committee. If no reprints are available, the titles and places of their publication are desired.

---

ALVARENGA PRIZE.—The College of Physicians of Philadelphia announces that the next award of the Alvarenga Prize, being the income for onc year of the bequest of the late Senor Alvarenga, and amounting to about $180, will be made on July 14, 1913, provided that an essay deemed by the Committee of Award worthy of the prize shall have been offered. Essays intended for competition may be upon any subject in medicine and must be unpublished. They must be typewritten in English or accompanied by an English translation, and received by the secretary of the college on or before May 1, 1913. Each essay must be sent without signature, but must be marked and accompanied by an envelope bearing the same mark and containing the name and address of the author. The successful essay or a copy of it remains the possession of the college.

---

SCIENTIFIC LESSON FROM THE ATTEMPTED ASSASSINATION OF COLONEL ROOSEVELT. Arthur MacDonald, a criminal anthropologist of Washington, and author of a Senate Document entitled "Man and Abnormal Man," suggests the establishment of laboratories or bureaus for the scientific investigation of criminals and other dangerous abnormals. He believes that every large city, and every State and especially the Federal Government, should have such a laboratory.

"When any one sends to the President, the governor, or mayor, or any prominent citizen threatening letters, or repeatedly utters threatening words, or attempts to injure such officials, or is unreasonably insistent in demanding to see them personally, such individual should be detained at least a few hours and thoroughly studied by scientific experts in criminal anthropology, psychophysics and social pathology."

A bill for such a laboratory is now pending before the Judiciary Committees of both Houses of Congress.

---

MALE DENTAL INTERNE. The U. S. Civil Service Commission announces an examination to be held Dec. 11, in various places throughout the country, including the following: (C. H. means Court House) Binghamton, Buffalo, Elmira, Ithaca, Jamestown, New York, C. H., Ogdensburg, C. H., Plattsburg, C. H., Poughkeepsie, Rochester, Syracuse, Troy and Utica.

There is at present one vacancy, in the Government Hospital for the Insane at Washington. No candidates presented themselves for the examination announced for Oct. 23. The salary is $600 and maintenance. Application should be made promptly for Examination form No. 1312.

---

MILK, IN BUFFALO, ADVANCED TO EIGHT CENTS A QUART. The hay crop this year is over 72 million tons, as against a little less than 55 million for 1911; there is also an unusually abundant late pasturage and a surplus of fresh vegetables beyond the demand for human consumption. Some of the milk dealers explain the rise in price as due to the increased severity of hygienic requirements; others say frankly that they want the additional profit. At any rate, unless the consumers can accomplish something, the cost of living will increase still further. The Housewives' League, organized in local circles has already attacked the problem with some success.

---

THE J. N. ADAM MEMORIAL HOSPITAL was dedicated on Tuesday, Nov. 12, at Perrysburg, N. Y. It is ideally located—save for its relative inaccessibility—on a hill side, 1500 feet above sea level, and only 50 feet lower than Raybrook. Beyond the expense of the site, which was donated by the late J. N. Adam during his mayoralty, it has cost about $285,000, $15,000 less than the allowance. It is adequate in every way but evinces a wholesome effort at economy throughout. The middle building, containing the dining room in a beautiful rotunda, with verandahs for use during summer, the kitchen, offices, recreation rooms, etc., connects with a dormitory building on each side, by corridors. While not quite completed, it is already in use and will accommodate about a hundred patients of both sexes. It is designed solely for incipient cases, a fact which the profession should remember in recommending patients. This limitation is necessary both for economy of maintenance, very few actual attendants being required, and to avoid the danger of infection and depressing influence of advanced cases, which will be cared for in the local county and projected municipal hospitals. Moreover, greater economy of construction could be secured than if the care of advanced cases, requiring more strict provisions for antisepsis and asepsis, had been contemplated.

Nearly two hundred physicians, philanthropists, politicians and near-politicians (to quote one of the speakers) attended the dedication. The generosity of the late J. N. Adam and the efficient services of Dr. John H. Pryor, were duly acknowledged, and in response, Dr. Pryor gave due credit to his associates and to the city officials, architects and contractors, all working in harmony.

The Hospital will be in charge of Dr. Otto R. Eichel, with Dr. Horace Lo Grasso, formerly of Buffalo, as assistant. Patients will pay for board so far as practicable but provision will also be made for suitable municipal charity cases. We understand that the thrifty Cattaraugus County assessors have already taxed the institution. It is a shame that any philanthropic institution should be taxed.

---

A Clinic on Poliomyelitis will be held at Alumni Hall, University of Buffalo, Fridays, Dec. 13 and 20, at 4 P. M. The profession is invited.

---

Sadistic Murder. The mysterious disappearance of a little boy from Lackawanna, last fall, has been cleared up by clues obtained from anonymous letters of confession, or rather of boasting, from a sexual pervert. The dismembered body was found in a vault. An arrest has been made but, at present writing, it is doubtful if the right person has been apprehended. Several similar crimes are referred to in the letters mentioned. Sexual perversion and inversion were rarely heard of in America up to a few years ago. Three inverts were known by reputation to Buffalo boys who are now middle-aged. One of these was a street car man of whose subsequent career, we are ignorant. The others were prominent merchants, both of whom are now dead. In the case of one of them, it is doubtful whether his inversion passed beyond the mental stage. The other may be considered as a facultative invert, as we heard complaints of his advances from two patients within a few days, one patient being a young man of fine physique, the other a woman. Unfortunately, within the last few years, disgusting practices have become so familiar that, a short time ago, we heard of a boy not over six years of age use a term of this nature in derogation of a playmate, probably in a figurative sense. Covert allusions are also occasionally heard on the stage and pervert practices are growing in frequency. No nation can persist in a state of indecency.

---

The Clinical Congress of Surgeons of North America held its third annual session in New York City, Nov 11 to 16, with Headquarters at the Waldorf-Astoria. The demonstrations included both operative general surgery and gynaecology, various surgical specialties, and radiology, also demonstrations of obstetric work, physiologic experiment, pathologic exhibits, etc. Evening lectures were held and various special trips, as to the Brooklyn Navy Yark, were arranged. As, for many of the clinics, special tickets were required, which were issued to applicants in order, at 8.30 A. M., it is obvious that the Congress involved steady hard work for the majority of those in attendance. About 2400 physicians were registered. Among the many useful accomplishments of the session, was the inauguration of a plan of lay teaching to secure early attention to malignant disease of the uterus. An unfortunate accident marred the occasion. A patient at the Polyclinic died, after the intraspinal injection of stovaine, before the operation, for hernia, by Dr. Wm. S. Brainbridge, could be begun.

